# Colistin Resistance in *Acinetobacter baumannii* MDR-ZJ06 Revealed by a Multiomics Approach

**DOI:** 10.3389/fcimb.2017.00045

**Published:** 2017-02-22

**Authors:** Xiaoting Hua, Lilin Liu, Youhong Fang, Qiucheng Shi, Xi Li, Qiong Chen, Keren Shi, Yan Jiang, Hua Zhou, Yunsong Yu

**Affiliations:** ^1^Department of Infectious Diseases, Sir Run Run Shaw Hospital, College of Medicine, Zhejiang UniversityHangzhou, China; ^2^Key Laboratory of Microbial Technology and Bioinformatics of Zhejiang ProvinceHangzhou, China; ^3^The Children's Hospital, College of Medicine, Zhejiang UniversityHangzhou, China; ^4^Centre of Laboratory Medicine, Zhejiang Provincial People's HospitalHangzhou, China; ^5^Hangzhou First People's HospitalHangzhou, China; ^6^Department of Respiratory, The First Affiliated Hospital, College of Medicine, Zhejiang UniversityHangzhou, China; ^7^State Key Laboratory for Diagnosis and Treatment of Infectious Diseases, Collaborative Innovation Center for Diagnosis and Treatment of Infectious Diseases, The First Affiliated Hospital, College of Medicine, Zhejiang UniversityHangzhou, China

**Keywords:** *Acinetobacter baumannii*, colistin, whole-genome sequencing, transcriptome, proteome

## Abstract

*Acinetobacter baumannii* has emerged as an important opportunistic pathogen due to its ability to acquire resistance to most currently available antibiotics. Colistin is often considered as the last line of therapy for infections caused by multidrug-resistant *A. baumannii* (MDRAB). However, colistin-resistant *A. baumannii* strain has recently been reported. To explore how multiple drug-resistant *A. baumannii* responded to colistin resistance, we compared the genomic, transcriptional and proteomic profile of *A. baumannii* MDR-ZJ06 to the induced colistin-resistant strain ZJ06-200P5-1. Genomic analysis showed that *lpxC* was inactivated by IS*Aba1* insertion, leading to LPS loss. Transcriptional analysis demonstrated that the colistin-resistant strain regulated its metabolism. Proteomic analysis suggested increased expression of the RND efflux pump system and down-regulation of FabZ and β-lactamase. These alterations were believed to be response to LPS loss. In summary, the *lpxC* mutation not only established colistin resistance but also altered global gene expression.

## Introduction

*Acinetobacter baumannii* has emerged as an important opportunistic pathogen due to its ability to acquire resistance to most currently available antibiotics (Peleg et al., 2008; Howard et al., 2012; Antunes et al., 2014). Since current treatment options for multi-drug resistant (MDR) *A. baumannii* are extremely limited, colistin is often considered as the last line of the therapy for infections caused by MDR *A. baumannii* (Bae et al., 2016; Cheah et al., 2016b). However, colistin-resistant *A. baumannii* strain has recently been reported (Cai et al., 2012).

Colistin is a polycationic antimicrobial peptide that targets the polyanionic bacterial lipopolysaccharide (LPS) of Gram-negative bacteria. Two different colistin resistance mechanisms have previously been reported (Beceiro et al., 2014). The first mechanism inactivates the lipid A biosynthesis pathway, leading to the complete loss of surface LPS. Mutations in *lpxC, lpxA*, or *lpxD* are involved in the first mechanism. The *pmrAB* two-component system mediates the second resistance mechanism. Mutations in *pmrA* and *pmrB* induce the activity of *pmrC*, which adds phosphoethanolamine (PEtn) to the hepta-acylated form of lipid A (Beceiro et al., 2011). Further mutations in *vacJ, pldA, ttg2C, pheS* and a conserved hypothetical protein were reported to involve in reduced colistin susceptibility through novel resistance mechanisms (Thi Khanh Nhu et al., 2016). Four putative colistin resistant genes: *A1S_1983, hepA, A1S_3026*, and *rsfS* were also identified in our previous study (Mu et al., 2016).

The response to LPS alteration has been investigated via transcriptional analysis. In response to LPS alteration, *A. baumannii* alters the expression of critical transport and biosynthesis systems associated with modulating the composition and structure of the bacterial surface (*lpxA*; Henry et al., 2012) or alters the expression of genes associated with outer membrane structure and biogenesis (*pmrB*; Cheah et al., 2016a). Moreover, the response to colistin is highly similar to the transcriptional alteration observed in an LPS-deficient strain (Henry et al., 2015). Colistin resistance was also explored using proteomic methods. There were 35 differentially expressed proteins. Most differentially expressed proteins were down-regulated in the colistin resistant strain, including outer membrane proteins, chaperones, protein biosynthesis factors, and metabolic enzymes (Fernandez-Reyes et al., 2009). However, the combination of genomic, transcriptomic, and proteomic methods to examine the colistin resistance mechanism in *A. baumannii* has rarely been reported. Furthermore, the strain used in this study was an MDR strain, but not laboratory strains (ATCC 19606, ATCC 17978) that do not represent clonal lineages in a clinical environment. Here, we used genome, transcriptome, and proteome to elucidate the colistin resistance mechanism in MDR *A. baumannii*. There was an ISAba1 insertion in *lpxC* (ABZJ_03720) in ZJ06-200P5-1 compared with the genome sequence of MDR-ZJ06, where *lpxC* encoded an UDP-3-O-acyl-N-acetylglucosamine deacetylase.

## Materials and methods

### Bacterial strains, media, and antibiotics

Restriction enzymes, T4 ligase, and Taq DNA polymerase were purchased from TaKaRa (Otsu, Shiga, Japan). The *A. baumannii* strain MDR-ZJ06 was isolated from the bloodstream of a patient in Hangzhou, China, in 2006. All *A. baumannii* cultures were grown at 37 °C in Mueller-Hinton (MH) agar and cation-adjusted MH broth (CAMHB) (Oxoid, Basingstoke, UK). Colistin was purchased from Sigma (Shanghai, China).

### Generation of colistin-resistant mutant

A colistin-resistant mutant was generated in *A. baumannii* MDR-ZJ06 by a previously described method (Li et al., 2006). Briefly, first, MDR-ZJ06 was cultured in CAMHB containing colistin at 8 × minimum inhibitory concentration (MIC). After overnight incubation, the culture was diluted 1:1000 with CAMHB containing colistin at 64 × MIC and then incubated at 37 °C overnight. Finally, the culture was diluted 1:100 with CAMHB containing colistin at 200 × MIC. After overnight incubation, the culture was plated on plates containing 10 μg of colistin at an appropriate dilution, and then one of colistin resistant colonies was collected for further experiments and designated as ZJ06-200P5-1. MICs for colistin and tigecycline were determined by *E*-test (bioMérieux, France) on MH agar, and the antimicrobial activities of the other antimicrobial agents were detected by disk diffusion. The results were interpreted according to CLSI or EUCAST breakpoints.

### Whole genome DNA sequencing and analysis

ZJ06-200P5-1 cells were cultured from a single colony overnight at 37 °C in MH broth. The genomic DNA was extracted via a QIAamp DNA minikit (Qiagen, Valencia, CA) following the manufacturer's protocol. Agarose gel and a NanoDrop spectrophotometer were used to determine the quality and quantity of extracted genomic DNA. The 300 bp library for Illumina paired-end sequencing was constructed from 5 μg of genome DNA of ZJ06-200P5-1 by staff at Zhejiang Tianke (Hangzhou, China). Mapping and SNP detection were performed via Breseq (Deatherage and Barrick, 2014). The regions containing the detected SNPs were amplified by PCR. The PCR products were sent to Biosune (Biosune, Hangzhou, China) for Sanger sequencing.

### Transcriptome analysis and real-time quantitative PCR verification

*A. baumannii* MDR-ZJ06 and ZJ06-200P5-1 were grown overnight at 37 °C in LB broth. Strains were subcultured 1/100 into fresh LB broth and grown at 37 °C for 2 h (OD_600_: 0.29 ± 0.02 for MDR-ZJ06, 0.26 ± 0.02 for ZJ06-200P5-1). The cells were collected at 4 °C, and the RNA was extracted using TRIZOL Reagent (Invitrogen, Carlsbad, CA, USA) after liquid nitrogen grinding. For RNA sequencing, wild type and mutants were sampled in triplicate. The subsequent RNA extraction, bacteria mRNA sequence library construction, transcriptome analysis and real-time quantitative PCR verification were performed by staff at Zhejiang Tianke (Hangzhou, China) as described previously in reference (Hua et al., 2014). Sequenced reads were mapped to the MDR-ZJ06 genome (CP001937-8) using Rockhopper (McClure et al., 2013). The output data was analyzed by edgeR (McCarthy et al., 2012). Data generated by RNA sequencing were deposited to the NCBI Sequence Read Archive with accession number SRR5234544 (the wild type) and SRR5234545 (the colistin resistant strain).

### Proteomic analysis

*A. baumannii* MDR-ZJ06 and ZJ06-200P5-1 were grown overnight at 37 °C in LB broth. Strains were subcultured 1/100 into fresh LB broth and grown at 37 °C for 2 h (OD_600_: 0.29 ± 0.02 for MDR-ZJ06, 0.26 ± 0.02 for ZJ06-200P5-1). The cells were collected at 4 °C and sent to Shanghai Applied Protein Technology Co. Ltd. The cell pellets were washed twice with PBS, and 500 μl SDT lysis buffer (4% SDS, 100 mM Tris-HCl, 1 mM DTT, pH 7.6) was added. After being sonicated for 2 mins on ice, the cells were centrifuged at 14,000 × g for 30 min at 4 °C. The protein concentration in the supernatant was determined by the BCA method.

In brief, 300 μg protein was added to 200 μl UA buffer (8 M urea, 150 mM Tris-HCl pH 8.0) and ultrafiltered (Sartorius, 10 kD) with UA buffer. To block reduced cysteine residues, 100 μl iodoacetamide (IAA) buffer (50 mM IAA in UA buffer) was added, centrifuged at 600 rpm for 1 min, and incubated for 30 min in the dark. The filter was washed twice with 100 μl UA buffer and twice with 100 μl Dissolution buffer (50 mM triethylammonium bicarbonate, pH 8.5). Finally, the proteins were digested with 2 μg trypsin (Promega) in 40 μl Dissolution buffer at 37 °C for 16–18 h. The peptides were collected as a filtrate, and its content was estimated at OD_280_.

For iTRAQ labeling, the peptides were labeled with the 4-plex iTRAQ reagent following the manufacturer's instructions (AB SCIEX). The peptides from MDR-ZJ06 were labeled with 114 and 116 isobaric reagents, and the peptides from ZJ06-200P5-1 were labeled with 115 and 117 isobaric reagents.

RP-HPCL online-coupled to MS/MS (LC-MS/MS) analysis of the iTRAQ-labeled peptides was performed on an EASY-nLC nanoflow LC system (Thermo Fisher Scientific) connected to an Orbitrap Elite hybrid mass spectrometer (Thermo Fisher Scientific). After the samples were reconstituted and acidified with buffer A (0.1% (v/v) formic acid in water), a set-up involving a pre-column and analytical column was used. The pre-column was a 2 cm EASY-column (100, 5 μm C18; Thermo Fisher Scientific), while the analytical column was a 10 cm EASY-column (75, 3 μm, C18; Thermo Fisher Scientific). The 120 min linear gradient from 0 to 100% buffer B (0.1% (v/v) formic acid and 80% acetonitrile) at a constant flow rate of 250 nl/min was as follows: 0–100 min, 0–35% buffer B; 100–108 min, 35–100% buffer B; 108–120 min, 100% buffer B. MS data were acquired using a data-dependent top 10 method, dynamically choosing the most abundant precursor ions from the survey scan (300–180 m/z) for HCD fragmentation. The Dynamic exclusion was set to a repeat count of 1 with a 30 s duration. Survey scans were acquired at a resolution of 30,000 at m/z 200, and the resolution for HCD spectra was set to 15,000 at m/z 200. The normalized collision energy was 35 eV, and the underfill ratio was defined as 0.1%.

The MS/MS spectra were searched using the MASCOT engine (Matrix Science, London, UK; version 2.2) against the *A. baumannii* MDR-ZJ06 FASTA database. False discovery rates (FDR) were calculated via running all spectra against the FASTA database using the MASCOT software. The following options were used to identify proteins: peptide mass tolerance = 20 ppm, fragment mass tolerance = 0.1 Da, Enzyme = Trypsin, Max missed cleavages = 2, Fixed modification: Carbamidomethyl (C), iTRAQ 4plex (N-term), iTRAQ 4plex (K), Variable modification: Oxidation (M). Quantification was performed based on the peak intensities of the reporter ions in the MS/MS spectra. The proteins were considered overexpressed when the iTRAQ ratio was above 1.5 and underexpressed when the iTRAQ ratio was lower than 0.67 (Wang et al., 2016). Functional classification of differentially expression genes were annotated using the KEGG databases. The mass spectrometry proteomics data have been deposited to the ProteomeXchange Consortium via the PRIDE (Vizcaino et al., 2016) partner repository with the dataset identifier PXD005265 and 10.6019/PXD005265. Reviewer account details: Username: reviewer54242@ebi.ac.uk; Password: zR8mE9wu.

### Growth rate determination

Four independent cultures per strain were grown overnight, diluted to 1:1000 in MH and aliquots placed into a flat-bottom 100-well plate in four replicates. The plate was incubated at 37 °C with agitation. The OD_600_ of each culture was determined every 5 min for 16 h using a Bioscreen C MBR machine (Oy Growth Curves Ab Ltd., Finland). The growth rate was estimated based on OD_600_ curves using an R script (Fang et al., 2016).

## Results

### Whole genome sequencing, minimum inhibitory concentration and growth rate

**Figure 1:**
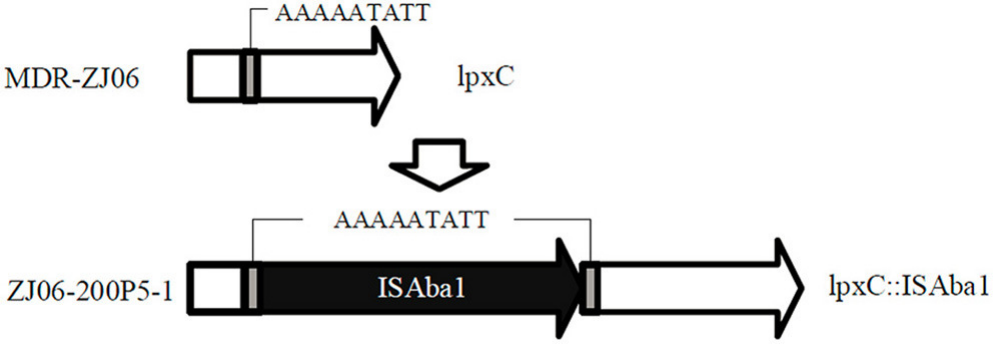
**Whole genome sequencing revealed the colistin-resistance mechanism in ***A. baumannii*** ZJ06-200P5-1**. The gene *lpxC* was intact in MDR-ZJ06, while in ZJ06-200P5-1, *lpxC* was inactivated by the insertion sequence ISAba1.

The colistin-resistant mutant ZJ06-200P5-1 generated from the culture in CAMHB containing colistin was sent for whole genome sequencing. There was an ISAba1 insertion in *lpxC* in ZJ06-200P5-1 compared with the genome sequence of MDR-ZJ06 (Figure 1). The MIC of MDR-ZJ06 and ZJ06-200P5-1 were detected and listed in Table 1. The MIC for colistin increased from 0.38 mg/L (MDR-ZJ06) to >256 mg/L (ZJ06-200P5-1). However, ZJ06-200P5-1 showed higher sensitivity to multiple antibiotics: β-lactams, carbapenem, tetracycline, and ciprofloxacin, but not aminoglycosides. Furthermore, ZJ06-200P5-1 showed a lower growth rate (0.81 ± 0.05) than wild type.

**Table 1:** **Antibiotic susceptibility of ***A. baumannii*** MDR-ZJ06 and its colistin resistant mutant ZJ06-200P5-1**.

**Strains**	**CO^a^**	**TGC^a^**	**IPM**	**MEM**	**FEP**	**CAZ**	**CTX**	**ATM**	**PRL**	**TZP**	**SCF**	**SAM**	**CN**	**AK**	**TE**	**MH**	**CIP**	**CT**
MDR-ZJ06	0.38 mg/L	4 mg/L	8	8	6	6	6	6	6	6	16	10	6	6	6	10	6	14
ZJ06-200P5-1	>256 mg/L	0.5 mg/L	22	22	20	20	15	22	17	19	30	22	6	6	8	26	9	6

*CO, colistin; TGC, tigecycline; IPM, imipenem; MEM, meropenem; FEP, cefepime; CAZ, ceftazidime; CTX, cefotaxime; ATM, aztreonam; PRL, Piperacillin; TZP, piperacillin/tazobactam; SCF, Cefoperazone/sulbactam; SAM, ampicillin/sulbactam; CN, gentamicin; AK, amikacin; TE, tetracycline; MH, minocycline; CIP, Ciprofloxacin; CT, colistin*.

a *The MIC of colistin and tigecycline were determined by broth dilution method, while antimicrobial sensitivity of other antibiotics were detected by disk diffusion*.

### Transcriptome analysis

**Figure 2:**
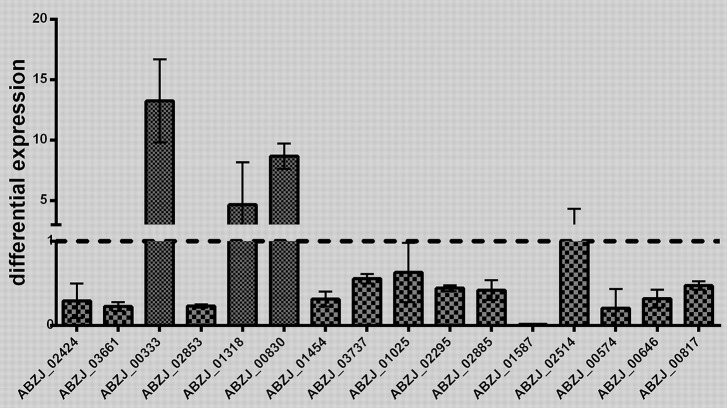
**Validation of the RNA sequencing results**. The transcriptomic results obtained by RNA-seq were validated by quantitative RT-PCR analysis. The differential expression of 16 genes was detected in this study. Three biology replicates were used in this experiment. The results were presented as expression in ZJ06-200P5-1, relative to MDR-ZJ06. The reference gene *rpoB* was used for inter-sample normalization. Error bars denote standard deviation.

The transcriptome analysis of ZJ06-200P5-1 and MDR-ZJ06 was performed by Illumina RNA deep sequencing technology. Cells of the two strains were collected in the early exponential phase. A total of 137 genes showed significant differential expression [log2(FoldChange) > 1 or log2(FoldChange) < −1], among which 48 genes were upregulated and 89 were downregulated (Table 2). Sixteen selected genes, three up-regulated and thirteen down-regulated genes, were well-validated by RT-qPCR (Figure 2). After mapping the differentially expressed genes into the KEGG pathway, we observed that genes involved in Energy metabolism and Amino acid metabolism were down-regulated, while Carbohydrate metabolism was up-regulated.

**Table 2:** **Genes changed significantly in transcriptome**.

**Synonym**	**Product**	**logFC**	**logCPM**	***P*****-value**	**FDR**
ABZJ_00055	hypothetical protein	8.308068	13.717	1.26E-78	4.54E-76
ABZJ_00068	hypothetical protein	6.4468	9.203574	2.14E-67	4.61E-65
ABZJ_00037	hypothetical protein	4.368832	9.669037	3.48E-68	9.36E-66
ABZJ_00056	hypothetical protein	4.349519	12.2059	6.03E-65	1.08E-62
ABZJ_00332	hypothetical protein	4.264896	9.455077	2.39E-53	2.86E-51
ABZJ_00036	hypothetical protein	3.449637	9.968726	9.61E-27	5.17E-25
ABZJ_01879	hypothetical protein	2.810666	6.769621	9.95E-35	7.65E-33
ABZJ_01880	putative transposase	2.758133	6.676606	5.52E-27	3.13E-25
ABZJ_01079	hypothetical protein	2.585295	6.001793	4.14E-10	6.55E-09
ABZJ_03753	hypothetical protein	2.318997	9.492231	2.51E-21	1.08E-19
ABZJ_00333	hypothetical protein	2.314205	5.437541	2.36E-11	4.53E-10
ABZJ_01881	transposase component	2.25458	8.338274	9.50E-21	3.93E-19
ABZJ_01133	heat shock protein	2.180889	13.35847	1.03E-25	5.06E-24
ABZJ_01180	putative phage-like protein	2.066152	3.22126	4.47E-06	3.56E-05
ABZJ_03752	PGAP1-like protein	2.014551	10.16569	2.49E-27	1.49E-25
ABZJ_00060	Thiol-disulfide isomerase and thioredoxin	1.894318	12.3252	7.68E-20	2.75E-18
ABZJ_00894	lactoylglutathione lyase-like protein	1.797874	6.779815	5.27E-15	1.62E-13
ABZJ_00054	N-alpha-acetylglutamate synthase (amino-acid acetyltransferase)	1.77044	10.25589	3.24E-20	1.27E-18
ABZJ_01151	hypothetical protein	1.634908	3.574211	4.88E-06	3.84E-05
ABZJ_03714	hypothetical protein	1.61859	8.500912	1.39E-08	1.85E-07
ABZJ_01900	acetoin:2,6-dichlorophenolindophenol oxidoreductase subunit alpha	1.527437	6.102611	2.98E-06	2.49E-05
ABZJ_01222	hypothetical protein	1.515854	2.111384	0.011897	0.034227
ABZJ_01191	hypothetical protein	1.46809	2.203352	0.011349	0.032877
ABZJ_01872	hypothetical protein	1.423713	7.613403	1.64E-08	2.10E-07
ABZJ_01187	hypothetical protein	1.423595	5.112417	2.82E-07	2.81E-06
ABZJ_01857	hypothetical protein	1.411761	2.566001	0.010144	0.029905
ABZJ_01829	Acyl-CoA dehydrogenase	1.402255	6.594396	4.45E-06	3.56E-05
ABZJ_01150	hypothetical protein	1.321675	3.205499	0.000936	0.003799
ABZJ_00028	lytic murein transglycosylase family protein	1.296752	10.96489	3.46E-14	9.79E-13
ABZJ_00976	hypothetical protein	1.295503	5.552053	1.46E-07	1.57E-06
ABZJ_01855	hypothetical protein	1.290522	2.587494	0.016132	0.044395
ABZJ_01186	hypothetical protein	1.249298	2.481015	0.013475	0.038054
ABZJ_00978	hypothetical protein	1.216859	3.038132	0.00684	0.021395
ABZJ_00977	hypothetical protein	1.209422	3.887522	0.000232	0.001118
ABZJ_00102	D-lactate dehydrogenase FAD-binding protein	1.170013	8.813908	1.91E-10	3.15E-09
ABZJ_01149	hypothetical protein	1.156232	3.314522	0.003302	0.011138
ABZJ_00053	alkanesulfonate transport protein	1.143156	6.421362	5.15E-06	3.99E-05
ABZJ_01275	hypothetical protein	1.122845	8.385252	1.31E-08	1.76E-07
ABZJ_03838	membrane-fusion protein	1.119324	7.708838	1.84E-08	2.33E-07
ABZJ_01901	acetoin:26-dichlorophenolindophenol oxidoreductase beta subunit	1.105826	6.349341	5.58E-05	0.000323
ABZJ_01899	lipoate synthase	1.08338	4.583472	0.003397	0.011422
ABZJ_00360	hypothetical protein	1.076106	8.065171	1.34E-07	1.46E-06
ABZJ_01210	hypothetical protein	1.065917	3.456549	0.011028	0.032156
ABZJ_01160	hypothetical protein	1.048988	3.144467	0.012194	0.034895
ABZJ_01148	hypothetical protein	1.048966	5.540519	1.77E-05	0.000122
ABZJ_00099	L-lactate permease	1.044891	10.0835	8.49E-08	9.61E-07
ABZJ_00901	major facilitator superfamily multidrug resistance protein	1.016944	9.235389	1.47E-08	1.91E-07
ABZJ_01775	6-pyruvoyl-tetrahydropterin synthase	1.014549	10.17374	3.05E-12	6.84E-11
ABZJ_03786	VirP protein	−1.0004	6.133241	3.35E-06	2.73E-05
ABZJ_01269	TPR repeat-containing SEL1 subfamily protein	−1.00222	4.702232	0.000305	0.001408
ABZJ_00120	hypothetical protein	−1.00591	7.042084	6.25E-07	5.85E-06
ABZJ_00896	nucleoside-diphosphate sugar epimerase	−1.0079	7.57903	9.80E-07	8.86E-06
ABZJ_01258	hypothetical protein	−1.01127	4.48134	0.002855	0.009692
ABZJ_01260	metal ion ABC transporter substrate-binding protein/surface antigen	−1.01249	9.488595	2.29E-08	2.86E-07
ABZJ_01120	urease accessory protein UreE	−1.01439	6.914944	6.34E-07	5.88E-06
ABZJ_01873	hypothetical protein	−1.01999	5.846082	1.89E-05	0.000128
ABZJ_03812	hypothetical protein	−1.02082	4.567471	0.001409	0.005227
ABZJ_01101	hypothetical protein	−1.03046	5.533349	0.001752	0.006282
ABZJ_01908	Zn-dependent hydrolase, including glyoxylase	−1.03588	9.460654	2.53E-10	4.12E-09
ABZJ_03819	hypothetical protein	−1.05745	9.905586	6.08E-11	1.11E-09
ABZJ_03796	putative acyltransferase	−1.06273	6.680253	2.34E-07	2.42E-06
ABZJ_00947	hypothetical protein	−1.0641	6.738813	1.36E-06	1.21E-05
ABZJ_01169	hypothetical protein	−1.06442	8.404764	8.75E-07	7.98E-06
ABZJ_00345	hypothetical protein	−1.06443	6.560939	2.47E-07	2.53E-06
ABZJ_03828	hypothetical protein	−1.06567	4.05012	0.000406	0.001813
ABZJ_00922	hypothetical protein	−1.07121	5.599955	7.64E-05	0.000424
ABZJ_01907	response regulator	−1.07682	6.813752	2.94E-07	2.90E-06
ABZJ_03790	gamma-aminobutyrate permease	−1.07931	8.18838	3.71E-05	0.000227
ABZJ_00882	hypothetical protein	−1.07943	9.751157	2.22E-11	4.34E-10
ABZJ_01078	hypothetical protein	−1.08109	10.14275	5.68E-14	1.49E-12
ABZJ_01132	glutamate dehydrogenase/leucine dehydrogenase	−1.08366	7.760303	2.14E-07	2.24E-06
ABZJ_03802	putative homogentisate 1,2-dioxygenase	−1.08726	6.643847	0.000162	0.000822
ABZJ_00334	hypothetical protein	−1.09533	6.571739	7.17E-08	8.25E-07
ABZJ_01250	outer membrane receptor protein	−1.10965	7.442322	0.000193	0.000956
ABZJ_00367	hypothetical protein	−1.11395	8.476819	9.04E-09	1.25E-07
ABZJ_00946	hypothetical protein	−1.12668	5.862006	7.32E-06	5.59E-05
ABZJ_01265	hypothetical protein	−1.12706	10.47521	4.03E-13	9.42E-12
ABZJ_01257	Zn-dependent protease with chaperone function	−1.13229	6.680195	1.30E-05	9.11E-05
ABZJ_01110	putative hemolysin-related protein	−1.13995	9.22038	1.74E-11	3.54E-10
ABZJ_03720	UDP-3-O-acyl-N-acetylglucosamine deacetylase	−1.14429	8.585685	1.05E-05	7.52E-05
ABZJ_01960	isochorismate hydrolase	−1.14761	5.633402	0.000121	0.000638
ABZJ_00942	hypothetical protein	−1.15912	8.72549	8.38E-09	1.17E-07
ABZJ_03859	putative RND type efflux pump involved in aminoglycoside resistance (AdeT)	−1.17363	8.75427	3.19E-05	0.000202
ABZJ_01874	hypothetical protein	−1.17434	5.206346	2.41E-05	0.000159
ABZJ_01917	putative acyl carrier protein phosphodiesterase (ACP phosphodiesterase)	−1.18991	7.045816	5.55E-08	6.50E-07
ABZJ_01861	membrane-fusion protein	−1.20577	6.002924	1.77E-07	1.87E-06
ABZJ_03742	hypothetical protein	−1.20817	3.772045	0.001579	0.005748
ABZJ_01262	hypothetical protein	−1.21556	4.167491	8.53E-05	0.000466
ABZJ_01929	Aspartate ammonia-lyase (Aspartase)	−1.21837	11.63816	9.24E-14	2.31E-12
ABZJ_00924	hypothetical protein	−1.2423	8.464578	1.01E-10	1.79E-09
ABZJ_01155	hypothetical protein	−1.2668	10.80427	1.20E-16	3.79E-15
ABZJ_00388	2-polyprenyl-6-methoxyphenol hydroxylase	−1.26695	7.901741	1.19E-09	1.81E-08
ABZJ_01862	multidrug ABC transporter ATPase	−1.27715	6.94382	4.78E-09	6.86E-08
ABZJ_00944	hypothetical protein	−1.28276	5.658916	2.33E-08	2.88E-07
ABZJ_01156	hypothetical protein	−1.28415	8.332979	5.92E-11	1.10E-09
ABZJ_01826	AraC-type DNA-binding domain-containing protein	−1.29289	5.11387	5.57E-07	5.26E-06
ABZJ_03744	hypothetical protein	−1.29678	8.720807	1.08E-08	1.47E-07
ABZJ_03737	hypothetical protein	−1.30269	10.28829	3.31E-20	1.27E-18
ABZJ_00940	hypothetical protein	−1.30722	6.280622	2.75E-07	2.79E-06
ABZJ_01218	hypothetical protein	−1.30837	4.257169	9.06E-06	6.63E-05
ABZJ_00061	putative transcriptional regulator	−1.31564	7.634498	1.67E-10	2.80E-09
ABZJ_01887	hypothetical protein	−1.3281	6.449578	1.02E-07	1.14E-06
ABZJ_01025	homocysteine/selenocysteine methylase	−1.33719	7.528478	3.07E-10	4.93E-09
ABZJ_00110	GNAT family acetyltransferase	−1.33942	4.887691	1.06E-06	9.50E-06
ABZJ_01242	hypothetical protein	−1.3506	7.369014	2.45E-09	3.61E-08
ABZJ_00895	hypothetical protein	−1.35351	6.693904	7.37E-12	1.56E-10
ABZJ_03712	putative flavoprotein	−1.38598	6.6067	2.04E-09	3.04E-08
ABZJ_00048	transcriptional regulator	−1.40027	7.755295	9.36E-11	1.68E-09
ABZJ_03785	glutamate racemase	−1.40496	7.417511	7.08E-12	1.52E-10
ABZJ_00938	hypothetical protein	−1.40799	6.629998	1.09E-10	1.88E-09
ABZJ_01230	hypothetical protein	−1.41279	10.19585	3.47E-19	1.20E-17
ABZJ_00124	glycine/D-amino acid oxidase (deaminating)	−1.46015	13.3987	8.58E-14	2.20E-12
ABZJ_03791	histidine ammonia-lyase (Histidase)	−1.49736	9.748038	2.37E-08	2.90E-07
ABZJ_03739	hypothetical protein	−1.49749	13.98113	3.54E-13	8.47E-12
ABZJ_00881	glutamine amidotransferase	−1.51327	8.144142	5.09E-14	1.37E-12
ABZJ_00988	hypothetical protein	−1.54819	6.1324	7.44E-09	1.05E-07
ABZJ_01840	putative ferric siderophore receptor protein	−1.55785	9.806018	9.74E-10	1.52E-08
ABZJ_00997	hypothetical protein	−1.58106	5.257799	3.12E-08	3.77E-07
ABZJ_00339	HSP90 family molecular chaperone	−1.6168	11.15864	7.57E-23	3.54E-21
ABZJ_00373	Type II secretory pathway, ATPase PulE/Tfp pilus assembly pathway, ATPase PilB	−1.6419	6.706339	3.45E-14	9.79E-13
ABZJ_01845	phosphatase/phosphohexomutase	−1.68301	7.222507	3.67E-12	8.06E-11
ABZJ_03793	urocanate hydratase	−1.69267	10.89217	1.13E-07	1.25E-06
ABZJ_03754	Rhs element Vgr family protein	−1.69503	8.757228	5.86E-18	1.97E-16
ABZJ_00945	hypothetical protein	−1.72533	5.192791	2.02E-11	4.03E-10
ABZJ_01002	putative ABC oligo/dipeptide transport, ATP-binding protein	−1.73182	6.449009	4.32E-14	1.19E-12
ABZJ_01259	hypothetical protein	−1.75565	7.198513	1.30E-12	2.98E-11
ABZJ_00114	short chain dehydrogenase family protein	−1.76754	7.176594	1.03E-13	2.52E-12
ABZJ_01177	hypothetical protein	−1.8053	8.135954	6.06E-15	1.81E-13
ABZJ_03792	hypothetical protein	−1.82418	6.284478	3.56E-06	2.88E-05
ABZJ_01219	hypothetical protein	−1.86448	9.22858	7.68E-22	3.45E-20
ABZJ_01088	carbonic anhydrase	−1.94984	9.430551	1.08E-27	6.83E-26
ABZJ_00346	hypothetical protein	−2.03948	6.219886	1.15E-16	3.73E-15
ABZJ_01207	hypothetical protein	−2.1746	7.126199	6.11E-20	2.27E-18
ABZJ_01886	hypothetical protein	−2.33548	5.458495	1.05E-11	2.18E-10
ABZJ_03766	putative secretory lipase precursor	−2.38284	9.073946	1.11E-31	7.47E-30
ABZJ_01206	hypothetical protein	−3.28101	9.194837	2.48E-45	2.42E-43
ABZJ_03736	thiol:disulfide interchange protein	−3.9361	9.872762	6.64E-41	5.50E-39

### iTRAQ

A total of 1582 proteins were identified in the iTRAQ experiment. A protein ratio >1.5 or <0.67 (*p* <0.05) was considered to be differentially expressed. After filtration, 82 differentially expressed proteins were identified between ZJ06-200P5-1 and MDR-ZJ06. The detailed information is shown in Table 3.

**Table 3:** **Genes changed significantly in proteome**.

**Protein number**	**NCBInr acession**	**Gene tag**	**Protein description**	**Pep Count**	**Unique PepCount**	**Coverage (%)**	**MW**	**pI**	**log2 of ratio (ZJ06-200P5-1 vs. MDR-ZJ06)**	***p*****-value**
233	384144952	ABZJ_03706	hypothetical protein	75	12	66.27	27649.89	4.59	1.65184	2.90E-20
1280	384143756	ABZJ_02510	hypothetical protein	1	1	10.18	17235.79	10.09	1.49121	8.79E-17
756	384144562	ABZJ_03316	hypothetical protein	27	4	34.13	13935.85	9.67	1.49075	8.99E-17
1032	384144568	ABZJ_03322	hypothetical protein	7	2	15.75	15550.26	10.03	1.39649	6.82E-15
565	384143898	ABZJ_02652	hypothetical protein	23	6	54.76	13282.22	8.99	1.15312	1.36E-10
594	384141430	ABZJ_00184	hypothetical protein	14	6	32.66	22273.87	4.56	1.131	3.05E-10
1241	384141854	ABZJ_00608	dehydrogenase	1	1	5.13	30137.1	8.79	1.11427	5.57E-10
1188	384141579	ABZJ_00333	hypothetical protein	5	1	10.66	11110.55	9.66	1.09309	1.18E-09
1076	384143755	ABZJ_02509	hypothetical protein	4	2	31.91	13701.31	10.29	1.09014	1.31E-09
147	384141823	ABZJ_00577	membrane-fusion protein	59	17	45.29	48231.1	9.44	0.9855	4.28E-08
1209	384142731	ABZJ_01485	dihydrodipicolinate synthase	2	1	2.89	33837.12	5.46	0.956837	1.05E-07
175	384143251	ABZJ_02005	membrane-fusion protein	50	15	47.22	43375.8	7.75	0.9115	4.13E-07
1281	384143760	ABZJ_02514	glycosyltransferase	1	1	3.37	48412.32	9.23	0.889123	7.92E-07
454	384141821	ABZJ_00575	putative outer membrane protein	18	8	21.57	54556.06	8.52	0.886277	8.60E-07
1009	384141578	ABZJ_00332	hypothetical protein	26	2	32.23	11005.53	9.93	0.859413	1.84E-06
1216	384143670	ABZJ_02424	hypothetical protein	2	1	25.58	4520.08	5.45	0.848157	2.51E-06
201	384143250	ABZJ_02004	cation/multidrug efflux pump	26	14	15.64	112744.8	7.6	0.801366	8.82E-06
885	384142076	ABZJ_00830	Outer membrane lipoprotein	12	3	18.75	21087.72	6.9	0.801241	8.85E-06
323	384144243	ABZJ_02997	putative porin protein associated with imipenem resistance	97	10	50.81	26505.22	4.8	0.770322	1.96E-05
1029	384141822	ABZJ_00576	peptide ABC transporter permease	7	2	3.77	71261.81	6.24	0.753391	2.98E-05
164	384144912	ABZJ_03666	NAD-dependent aldehyde dehydrogenase	41	16	43.15	51846.55	5.11	0.751721	3.11E-05
655	384144155	ABZJ_02909	hypothetical protein	27	5	33.48	26172.15	7.85	0.733875	4.80E-05
812	384142146	ABZJ_00900	multidrug resistance secretion protein	8	4	9.14	40956.99	6.56	0.691132	0.000131
852	384145008	ABZJ_03762	putative short-chain dehydrogenase	6	4	17.24	31854.29	9.26	0.688359	0.000139
539	384144680	ABZJ_03434	flavoprotein	10	7	15.52	55720.24	9.12	0.685088	0.00015
150	384144913	ABZJ_03667	4-aminobutyrate aminotransferase	55	17	50.23	45976.96	5.81	0.679784	0.000169
1306	384144561	ABZJ_03315	kinase sensor component of a two component signal transduction system	1	1	3.07	62690.76	6.3	0.672652	0.000198
600	384144948	ABZJ_03702	xenobiotic reductase	14	6	21.02	38725.16	5.08	0.608194	0.000783
315	384144930	ABZJ_03684	hypothetical protein	322	10	47.37	32732.07	4.71	0.602647	0.000876
603	384143541	ABZJ_02295	UDP-glucose 4-epimerase	13	6	28.06	38064.02	5.53	0.599175	0.000939
384	384142564	ABZJ_01318	Zn-dependent protease with chaperone function	35	9	48.66	27572.18	9.44	0.592971	0.001063
680	384143417	ABZJ_02171	hypothetical protein	14	5	40.65	17046.41	8.79	−0.59205	0.000855
996	384143586	ABZJ_02340	hypothetical protein	3	3	10.61	29941.63	6.85	−0.60757	0.000626
1007	384145105	ABZJ_03859	putative RND type efflux pump involved in aminoglycoside resistance (AdeT)	3	3	10.48	38641.56	9.71	−0.60779	0.000623
667	384144990	ABZJ_03744	hypothetical protein	18	5	21.99	27747.62	4.62	−0.60878	0.00061
820	384141318	ABZJ_00072	FKBP-type 22KD peptidyl-prolyl cis-trans isomerase	7	4	21.65	25217.38	9.06	−0.61264	0.000564
767	384141553	ABZJ_00307	hypothetical protein	17	4	48.31	10746.92	5.3	−0.61297	0.00056
163	384144907	ABZJ_03661	hypothetical protein	47	16	39.91	49757.27	8.16	−0.61374	0.000551
865	384144338	ABZJ_03092	Zn-dependent hydrolase, including glyoxylase	5	4	15.00	35333.86	8.91	−0.62839	0.000407
780	384141775	ABZJ_00529	gluconate kinase	12	4	30.59	18924.48	4.88	−0.6352	0.000353
1259	384142716	ABZJ_01470	hypothetical protein	1	1	2.52	36304.38	9.04	−0.63588	0.000348
424	384142064	ABZJ_00818	3-oxoacyl-ACP reductase	42	8	45.90	26098.39	6.1	−0.64296	0.000299
825	384141812	ABZJ_00566	hypothetical protein	7	4	36.11	15329.44	9.46	−0.64431	0.00029
381	384141306	ABZJ_00060	Thiol-disulfide isomerase and thioredoxin	37	9	42.44	22825.09	9.58	−0.65529	0.000229
963	384142833	ABZJ_01587	dehydrogenase	4	3	9.93	31970.72	5.16	−0.6827	0.000125
645	384141583	ABZJ_00337	putative outer membrane protein W	52	5	28.64	22680.64	5.9	−0.69549	9.35E-05
329	384142063	ABZJ_00817	malonyl-CoA-[acyl-carrier-protein] transacylase	59	10	43.15	35339.2	5.22	−0.6997	8.49E-05
941	384142271	ABZJ_01025	homocysteine/selenocysteine methylase	5	3	12.33	32062.1	4.82	−0.71762	5.59E-05
716	384144502	ABZJ_03256	protein-disulfide isomerase	9	5	23.31	26361.06	9	−0.72106	5.15E-05
232	384144545	ABZJ_03299	acetylCoA carboxylase subunit beta	76	12	44.63	32971.73	5.85	−0.72297	4.93E-05
836	384144135	ABZJ_02889	hypothetical protein	7	4	38.57	15413.52	8.43	−0.72309	4.91E-05
207	384141892	ABZJ_00646	Acetyl-CoA carboxylase alpha subunit	87	13	75.09	29640.53	5.6	−0.72798	4.37E-05
1053	384144131	ABZJ_02885	LysR family transcriptional regulator	5	2	6.80	34516.26	6.26	−0.74843	2.67E-05
883	384142465	ABZJ_01219	hypothetical protein	14	3	26.54	17636.93	9.58	−0.75975	2.02E-05
791	384142700	ABZJ_01454	hypothetical protein	10	4	25.15	19116.5	5	−0.77251	1.47E-05
573	384144158	ABZJ_02912	putative fatty acid desaturase	20	6	17.03	42202.21	9.39	−0.77608	1.34E-05
663	384141673	ABZJ_00427	putative type III effector HopPmaJ	19	5	37.27	12074.21	5.41	−0.78501	1.07E-05
261	384141776	ABZJ_00530	NAD-dependent aldehyde dehydrogenase	28	12	22.69	60150.9	6.04	−0.80138	7.02E-06
166	384141820	ABZJ_00574	NADH-dependent enoyl-ACP reductase	142	15	64.24	31016.41	6	−0.81807	4.53E-06
280	384144728	ABZJ_03482	putative toluene tolerance protein (Ttg2D)	76	11	61.97	23513.33	9.83	−0.82764	3.51E-06
917	384142976	ABZJ_01730	hypothetical protein	7	3	14.80	21011.63	9.2	−0.8625	1.36E-06
192	384144009	ABZJ_02763	hypothetical protein	63	14	48.19	44493.93	8.79	−0.86539	1.25E-06
635	384144826	ABZJ_03580	putative penicillin binding protein (PonA)	8	6	8.23	94767.31	9.38	−0.88231	7.77E-07
292	384142962	ABZJ_01716	biotin synthetase	40	11	34.83	37136.95	5.45	−0.89634	5.20E-07
909	384142828	ABZJ_01582	putative 17 kDa surface antigen	8	3	44.76	12431.23	4.7	−0.93167	1.85E-07
483	384144247	ABZJ_03001	hypothetical protein	43	7	48.55	14704.84	9.54	−0.93498	1.67E-07
188	384142100	ABZJ_00854	beta-ketoacyl-ACP synthase	90	14	46.45	43130.17	5.2	−0.94675	1.17E-07
446	384144999	ABZJ_03753	hypothetical protein	22	8	39.09	28038.81	9.07	−0.95428	9.33E-08
401	384144159	ABZJ_02913	flavodoxin reductase (ferredoxin-NADPH reductase) family protein 1	23	9	31.46	39570.7	6.09	−0.95498	9.13E-08
489	384143515	ABZJ_02269	(3R)-hydroxymyristoyl-ACP dehydratase	39	7	50.93	17988.69	6.3	−0.97767	4.53E-08
459	384142835	ABZJ_01589	hypothetical protein	18	8	13.33	43721.35	4.96	−0.97866	4.39E-08
833	384143336	ABZJ_02090	hypothetical protein	7	4	37.91	17951.03	4.82	−1.00115	2.16E-08
586	384143810	ABZJ_02564	hypothetical protein	16	6	79.22	8718.62	5	−1.02063	1.15E-08
114	384143236	ABZJ_01990	beta-lactamase OXA-23	161	18	71.38	31385.05	8.37	−1.0965	8.98E-10
359	384143517	ABZJ_02271	putative outer membrane protein (OmpH)	25	10	57.49	18710.09	9.52	−1.22331	8.58E-12
606	384144983	ABZJ_03737	hypothetical protein	13	6	38.04	27580.52	4.68	−1.28419	7.75E-13
95	384144431	ABZJ_03185	putative DcaP-like protein	111	20	50.69	47278.17	6.37	−1.36068	3.23E-14
939	384141906	ABZJ_00660	putative lipoprotein precursor (VacJ) transmembrane	5	3	10.67	33499.98	4.85	−1.43792	1.09E-15
1289	384144099	ABZJ_02853	hypothetical protein	1	1	8.06	14811.21	4.39	−1.58122	1.26E-18
1186	384142065	ABZJ_00819	acyl carrier protein (ACP)	21	1	10.99	10132.23	4.11	−1.65109	3.70E-20
412	384142699	ABZJ_01453	hypothetical protein	14	9	46.52	25412.88	9.89	−1.66497	1.81E-20
113	384144004	ABZJ_02758	beta-lactamase	268	18	55.05	44683.92	9.28	−1.82062	3.88E-24

**Figure 3:**
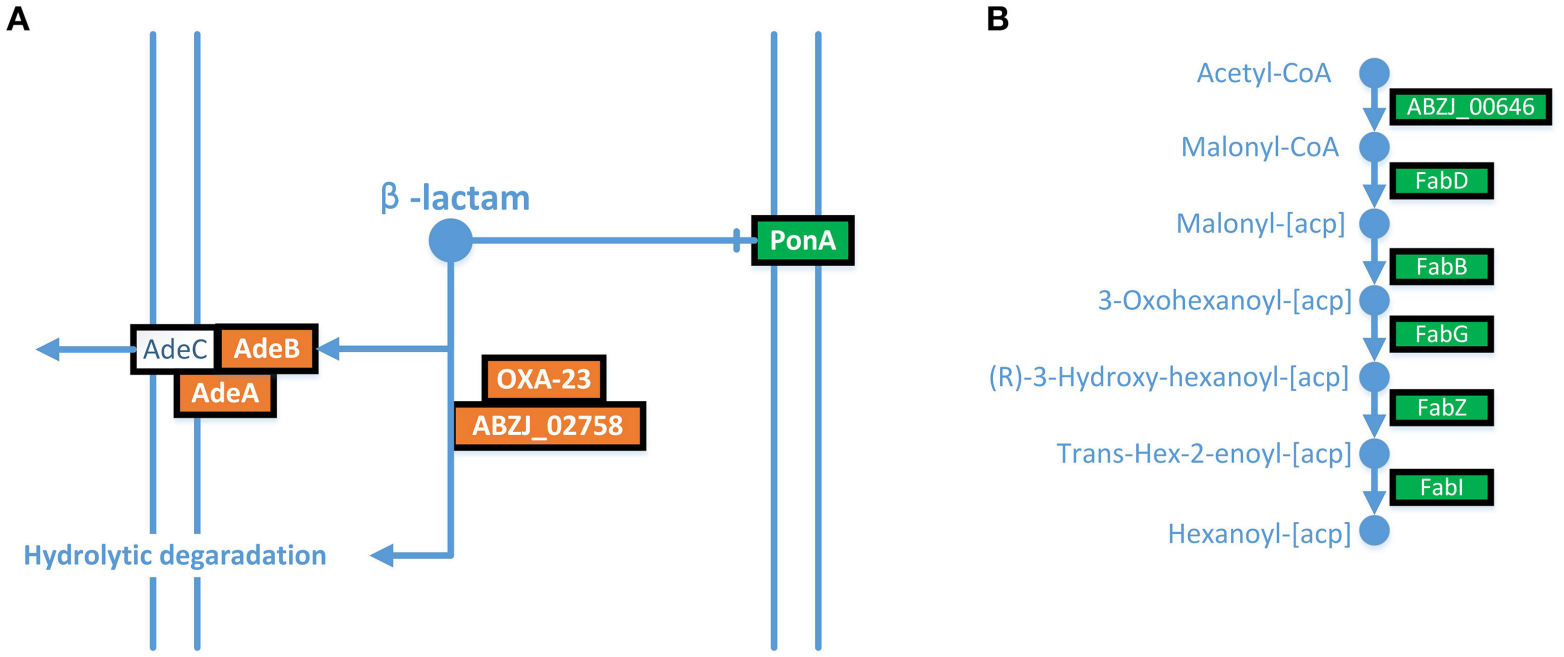
**ITRAQ analysis showed that AdeABC were up-regulated, and the fatty acid biosynthesis pathway was down-regulated in ZJ06-200P5-1. (A)** AdeABC efflux pump, **(B)** fatty acid biosynthesis pathway. Green shows genes with significantly reduced expression levels, and red shows genes with significantly increased expression levels.

The expression of AdeABC was up-regulated in the LPS-loss ZJ06-200P5-1 strain. The AdeABC efflux pump confers resistance to various antibiotics classes. The expression of AdeABC genes was increased approximately two-fold in ZJ06-200P5-1 (Figure 3A). However, ZJ06-200P5-1 showed higher susceptibility to multiple antibiotics than MDR-ZJ06 (Table 1).

The fatty acid biosynthesis pathway was down-regulated in the ZJ06-200P5-1 strain (Figure 3B). The expression of FabZ was decreased by approximately two-fold in ZJ06-200P5-1. The β-lactamases *bla*_OXA−23_ and *bla*_ADC−25_ were down-regulated in ZJ06-200P5-1 strain. The expression levels of *bla*_OXA−23_ and *bla*_ADC−25_ were decreased two- to four-fold in ZJ06-200P5-1.

### Common genes altered expression in both transcriptome and proteome

A total of 15 differentially expressed genes (or proteins) were identified in both transcriptome and proteome (Table 4). Among them, three genes were both up-regulated, and nine genes were both down-regulated. Although there was correlation between transcriptome and proteome data, the absolute expression difference values in transcriptome data was higher than those in proteome data. In addition, the result of three gene/proteins were contradictory (highlighted in red letters in Table 4). The contradictory result might be caused by post-transcriptional regulation.

**Table 4:** **Common genes altered expression both in transcriptome and proteome**.

**Synonym**	**Product**	**Fold change (log2, Transcriptome)**	**Fold change (log2, Proteome)**
ABZJ_00332	hypothetical protein	4.26489563	0.859413
ABZJ_03753	hypothetical protein	2.318997325^a^	−0.95428
ABZJ_00333	hypothetical protein	2.314204886	1.09309
ABZJ_01133	heat shock protein	2.180888936	0.532117
ABZJ_00060	Thiol-disulfide isomerase and thioredoxin	1.894317881^a^	−0.65529
ABZJ_00028	lytic murein transglycosylase family protein	1.296751692^a^	−0.57293
ABZJ_01078	hypothetical protein	−1.081092562	−0.44448
ABZJ_03720	UDP-3-O-acyl-N-acetylglucosamine deacetylase	−1.144287283	−0.48378
ABZJ_03859	putative RND type efflux pump involved in aminoglycoside resistance (AdeT)	−1.173634714	−0.60779
ABZJ_03744	hypothetical protein	−1.296782077	−0.60878
ABZJ_03737	hypothetical protein	−1.302692756	−1.28419
ABZJ_01025	homocysteine/selenocysteine methylase	−1.337189269	−0.71762
ABZJ_01219	hypothetical protein	−1.864476303	−0.75975
ABZJ_01088	carbonic anhydrase	−1.949843631	−0.56001
ABZJ_01206	hypothetical protein	−3.281014801	−0.4346

a *The result of three gene/proteins were contradictory*.

## Discussion

Due to the limitation of antimicrobial agents in clinical use, it is urgent to extend our understanding of the emergence of colistin resistance in *A. baumannii*. *A. baumannii* MDR-ZJ06, a multidrug-resistant clinical strain isolated from bloodstream, has been sequenced and was considered an ideal strain for examining the colistin-resistant mechanism in *A. baumannii* (Zhou et al., 2011). In this study, colistin-resistant strain was rapidly obtained, and its resistance mechanism was LPS loss caused by IS*Aba1* insertion in *lpxC*. This result confirmed a previous finding (Moffatt et al., 2010). The rapid isolation of colistin-resistant mutant from multiple drug-resistant *A. baumannii* indicated a high risk of *A. baumannii* evolving resistance to colistin in clinical use.

We successfully detected the whole transcriptional profile of *A. baumannii* strain MDR-ZJ06 and its colistin-resistant mutant ZJ06-200P5-1 via Illumina RNA-sequencing. In another transcriptome study (Henry et al., 2012), *A. baumannii* ATCC 19606 and its *lpxA* mutant were used. Although both the *lpxC* and *lpxA* mutation lead to LPS loss, the different transcriptional response may be due to differences in the strain genetic background and the resistant mutation. In transcriptional analysis, we observed that genes involved in Energy metabolism and Amino acid metabolism were down-regulated, while Carbohydrate metabolism was up-regulated.

The expression of AdeABC was up-regulated in the LPS-loss ZJ06-200P5-1 strain. Similar results were also observed in all polymyxin-treated samples (Cheah et al., 2016a). In addition, the expression levels of *adeIJK* and *macAB-tolC* were up-regulated in the LPS loss mutant (Henry et al., 2012). Increased expression of the RND efflux pump system (AdeABC) was a common finding across all experiments in colistin exposure. The up-regulation of AdeABC indicated the diminished integrity and barrier function of the outer membrane in colistin-resistant *A. baumannii* (Henry et al., 2015; Cheah et al., 2016a). However, ZJ06-200P5-1 showed higher susceptibility to multiple antibiotics than MDR-ZJ06. The higher susceptibility might result from the higher outer membrane permeability of ZJ06-200P5-1 due to LPS-loss. The increased expression of the efflux pump was thought to be a response to toxic substances that accumulated in the cells due to the increased membrane permeability (Henry et al., 2012).

The fatty acid biosynthesis pathway was down-regulated in the ZJ06-200P5-1 strain. In *E. coli*, it is important to balance LPS and fatty acid biosynthesis to maintain cell integrity. FabZ, which dehydrates R-3-hydroxymyristoyl-acyl carrier protein in fatty acid biosynthesis, plays an important role in rebalancing lipid A and fatty acid homeostasis (Bojkovic et al., 2016). The decrease in FabZ was considered to be a response to LPS-loss in ZJ06-200P5-1. The β-lactamases *bla*_OXA−23_ and *bla*_ADC−25_ were down-regulated in the ZJ06-200P5-1 strain. Decreased expression levels of *bla*_OXA−23_ and *bla*_ADC−25_ were also observed in *A. baumannii* MDR-ZJ06 under a subinhibitory concentration of tigecycline (Hua et al., 2014). Meanwhile, the strain under tigecycline stress showed a lower MIC of ceftazidime (Hua et al., 2014). The decrease in *bla*_OXA−23_ and *bla*_ADC−25_ might contribute to the increased sensitivity to β-lactam antimicrobial agents.

A multi-omics approach was adopted to obtain a more global view of colistin-resistant *A. baumannii*. Genomic analysis showed that *lpxC* was inactivated by IS*Aba1* insertion, leading to LPS loss. Transcriptional analysis demonstrated that the colistin-resistant strain regulated its metabolism. Metabolic change and LPS loss were concomitant. Proteomic analysis suggested increased expression of the RND efflux pump system and the down-regulation of FabZ and β-lactamase. These alterations are believed to be responses to LPS loss. Together, the *lpxC* mutation not only confirmed colistin resistance but also altered global gene expression.

### Nucleotide sequence accession numbers

The whole-genome shotgun sequencing results for *A. baumannii* ZJ06-200P5-1 have been deposited at DDBJ/EMBL/GenBank under the accession number MIFW00000000.

## Author contributions

XH and YY conceived and designed the study. XH, LL, YF, QS, XL, QC, KS, YJ, and HZ performed the experiments. XH and YY performed data analysis and drafted the manuscript. All authors reviewed and approved the final manuscript.

### Conflict of interest statement

The authors declare that the research was conducted in the absence of any commercial or financial relationships that could be construed as a potential conflict of interest.
